# Supplementary material for: Correlates of protection for booster doses of the SARS-CoV-2 vaccine BNT162b2
Source: Nat Commun. 2023 Jul 29;14:4575. doi: 10.1038/s41467-023-39816-4 (PMC10387073; doi:10.1038/s41467-023-39816-4)
Supplement: Supplementary file 1 — Supplementary Information [file 41467_2023_39816_MOESM1_ESM.pdf]

Title: Correlates of protection for booster doses of the SARS-CoV-2 vaccine BNT162b2

Authors: Tomer Hertz[1,2,3*], Shlomia Levy[1,2]†, Daniel Ostrovsky[4]†, Hannah Oppenheimer[1,2]†, Shosh Zismanov[1,2]†, Alona Kuzmina[1]†, Lilach M Friedman[1,2], Sanja Trifkovic[5], David Brice[6], Lin Chun-Yang[6], Liel, Cohen-Lavi[1,2], Yonat Shemer-Avni[1,7], Merav Cohen-Lahav[8], Doron Amichay[9], Ayelet Keren-Naus[1,7], Olga Voloshin[7], Gabriel Weber[10,11], Ronza Najjar-Debbiny[10,11], Bibiana Chazan[11,12], Maureen A. McGargill[6], Richard Webby[5], Michal Chowers[13,14], Lena Novack[4], Victor Novack[4], Ran Taube[1*], Lior Nesher[15*], Orly Weinstein[16,17].

Affiliations:

[1]Department of Microbiology and Immunology, Faculty of Health Sciences, Ben-Gurion University of the Negev, Beer-Sheva, Israel.
[2]National Institute of Biotechnology in the Negev, Ben-Gurion University of the Negev, Beer-Sheva, Israel.
[3]Fred Hutch Cancer Research Center, Seattle, WA, USA.
[4]Clinical Research Center, Soroka University Medical Center, and the faculty of Health Sciences, BenGurion University of the Negev, Beer-Sheva, Israel
[5]Department of Infectious Diseases, St. Jude Children's Research Hospital, Memphis, TN, USA
[6]Department of Immunology, St. Jude Children's Research Hospital, Memphis, TN, USA [7]Laboratory of Virology, Soroka University Medical Center, Beer-Sheva, Israel.
[8]Laboratory of Management, Soroka University Medical Center, Beer-Sheva, Israel.
[9]Central Laboratory, Clalit Health Services & Dept. of Clinical Biochemistry and Pharmacology, Faculty of Health Sciences, Ben-Gurion University of the Negev, Beer Sheba, Israel.
[10]Infectious Diseases Unit, Lady Davis Carmel Medical Center, Haifa, Israel.
[11]Rappaport Faculty of Medicine, Technion-Israel Institute of Technology, Haifa, Israel.
[12]Infectious Diseases Unit, Emek Medical Center, Afula, Israel.
[13] School of Medicine, Tel Aviv University, Tel Aviv, Israel.
[14] Meir Medical Center, Kfar Saba, Israel.
[15]Infectious Disease Institute, Soroka University Medical Center, and Faculty of Health Sciences, BenGurion University, Beer Sheba, Israel.
[16]Dept. of Health systems management, faculty of health sciences, Ben-Gurion University of the Negev, Beer Sheva, Israel.
[17]Hospital division, Clalit Health Services, Tel Aviv, Israel

* Corresponding authors
† These authors contributed equally

These authors contributed equally: Shlomia Levy, Daniel Ostrovsky, Hannah Oppenheimer, Shosh Zismanov, Alona Kuzmina Correspondence and requests for materials should be addressed to T.H (email: thertz@post.bgu.ac.il), R.T (email: rantaube@bgu.ac.il), L.N (email: nesherke@bgu.ac.il) These authors jointly supervised this work: Tomer Hertz, Ran Taube and Lior Nesher

Supplementary Materials

Supplementary Figs. 1 to 5

Supplementary Tables 1 to 16

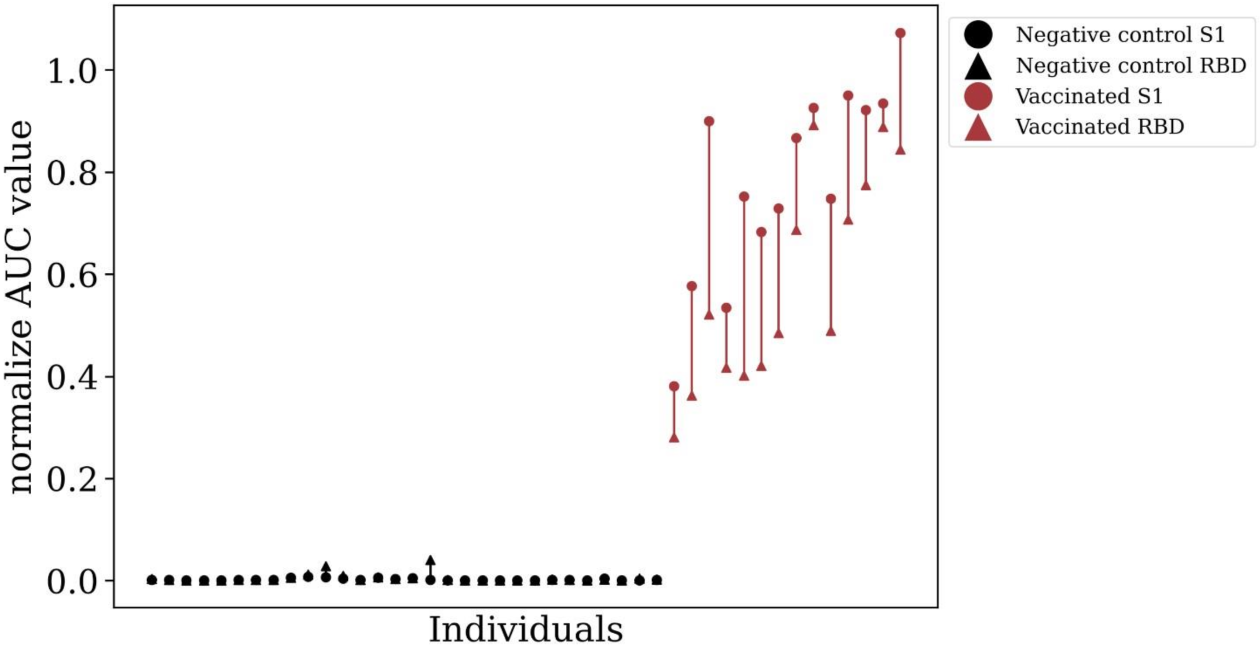

Supplementary Fig. 1: Comparing SARS-CoV-2 antibody levels in pre-pandemic and pandemic serum samples. Normalized AUC IgG antibody levels to the S1 and receptor binding domain (RBD) proteins of SARS-CoV-2 Wuhan strain were measured using an antigen microarray. Negative control samples of 30 individuals collected prior to the SARS-CoV-2 pandemic were compared with 14 individuals that received 3 doses of the Pfizer-Biontech BNT162b2 vaccine. IgG AUC levels were computed across 6 antigen concentrations (2.03 µg/mL - 65 µg/mL). A two sided Wilcoxon ranksum test was used to compare the two groups. p value < 0.0001

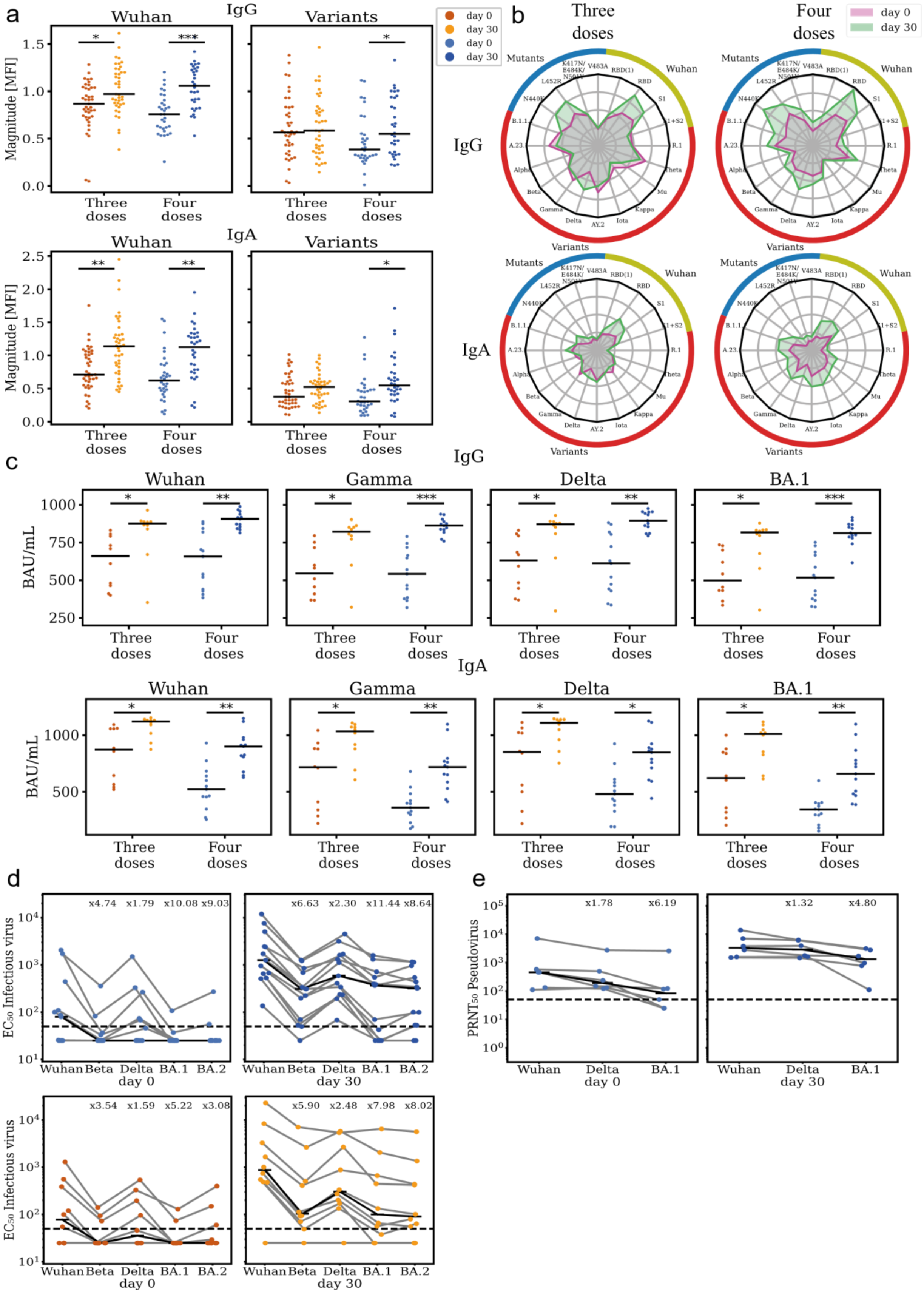
a
IgG
Wuhan
Variants
Magnitude [MFI]
Three doses
Four doses
IgA
day 0
day 30
b
Three doses
Four doses
Mutants
Wuhan
Variants
K417N/ E484K/ N501Y
V483A
RBD(1)
RBD
S1
S1+S2
R.1
Theta
Mu
Kappa
Iota
AY.2
Delta
Gamma
Beta
Alpha
A.23.
B.1.1.
N440K
L452R
c
Gamma
Delta
BA.1
BAU/mL
d
x4.74 x1.79 x10.08 x9.03
x6.63 x2.30 x11.44 x8.64
x3.54 x1.59 x5.22 x3.08
x5.90 x2.48 x7.98 x8.02
EC50 Infectious virus
Wuhan Beta Delta BA.1 BA.2
day 0
day 30
e
x1.78 x6.19
x1.32 x4.80
PRNT50 Pseudovirus
Wuhan Delta BA.1

Supplementary Fig. 2: Infection with omicron elicited binding and neutralizing antibodies against SARS-CoV-2. Responses of individuals that were infected with omicron within the first 30 days after enrollment were analyzed at enrollment (day 0) and at day 30 using multiple serological assays. Individuals that received three (n=41) or four doses (n=32) of the vaccine were analyzed separately. **a** IgG and IgA magnitude to antigens from the Wuhan strain and SARS-COV-2 variants. Antigen microarrays spotted with receptor binding domain (RBD), S1 and spike proteins of the Wuhan vaccine strain and multiple other variants of concern were used to measure the magnitude of responses at day 0 (enrollment) and day 30 post enrollment. Black lines denote the median. P-values were computed using the two-sided wilcoxon ranksum test. * $p < 0.05$; ** $p < 0.001$; *** $p < 0.0001$. **b** Spider plots depicting the enrollment (pink) and day 30 (green) antibody levels to Wuhan antigens (gold), variants of concern (red) and RBD mutants (blue). The average normalized magnitude to each antigen is plotted in individuals that received three or four doses. **c** IgG and IgA anti RBD ELISA binding titers for a subset of 74 participants. **d** infectious virus neutralization half maximal effective concentration (EC50) titers of the same individuals in panel c. **e** Pseudovirus neutralization titers of uninfected individuals that received four doses (n=13, blue).

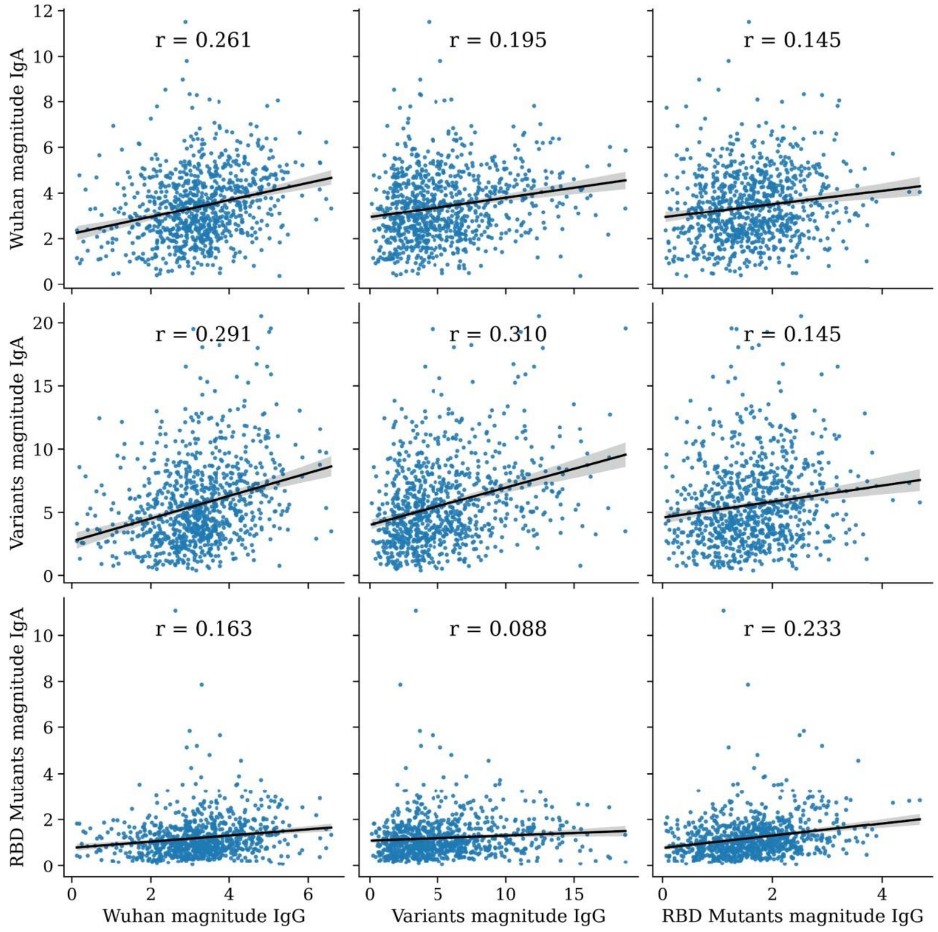

Supplementary Fig. 3: Correlation between IgG and IgA selected markers. The correlation between the IgG and IgA markers that were used for combinations are presented. We used Pearson correlation coefficient to assess the association between the IgG and IgA markers. Black lines represent the linear regression model fit, and gray band line denotes the 95% confidence intervals.

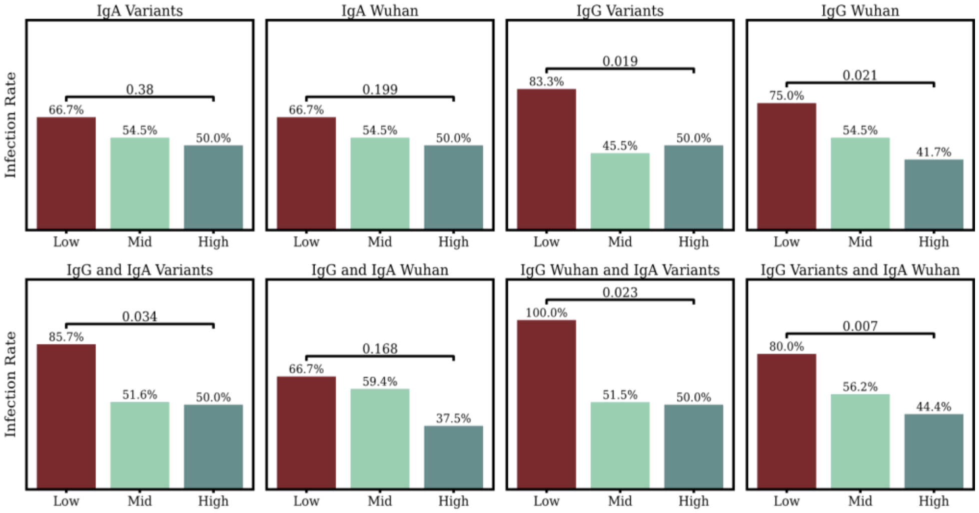

b

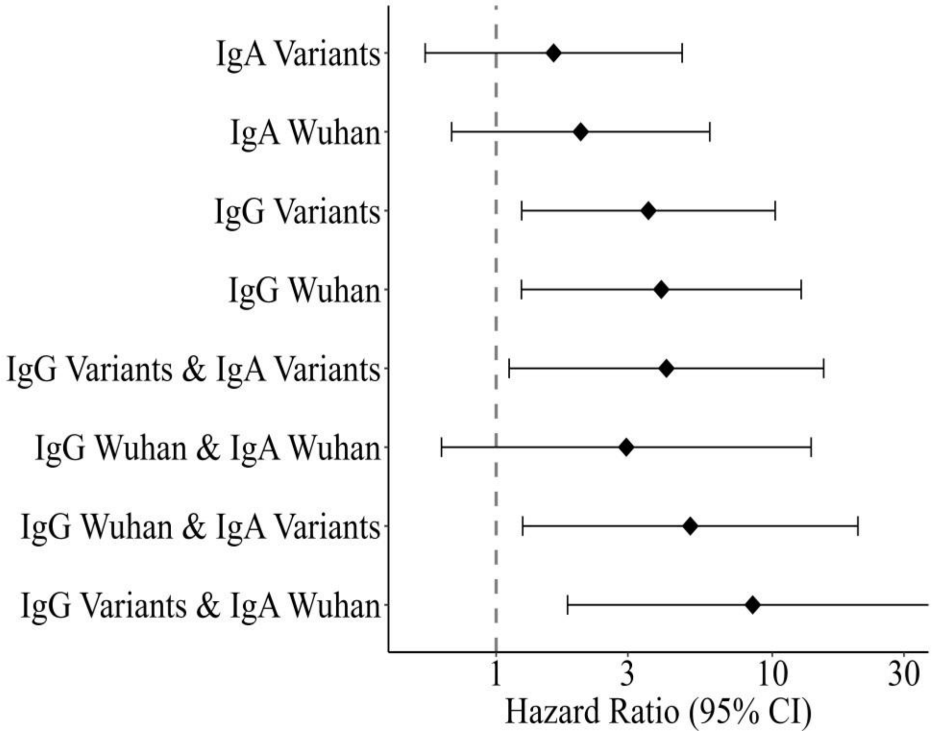

Supplementary Fig. 4: Baseline correlates of protection in a validation cohort at an interim timeopint. An independent cohort of 46 individuals was followed for 290 days. Individuals were ranked by several baseline binding antibody markers into low- mid- and high response groups, and SARS-CoV-2 infection rates of each group were compared at day 198 at the end of the omicron wave in Israel (April 2022). **a** Infection rates in the low- mid- and high-baseline response groups based on: IgA magnitude to SARS-CoV2

variants, IgA magnitude to Wuhan, IgG magnitude to SARS-CoV-2 variants, IgG magnitude to Wuhan (top row) and their combinations (bottom row). P-values were computed using a cox proportional hazard model, adjusted for age, sex and number of vaccine doses. **b** Hazard ratios for the four primary baseline markers (n=24) and their combinations comparing low to high baseline response groups (n=13-15). The dot represents the hazard ratios, error bars denote the 95% confidence intervals. Hazard ratios were computed using a cox proportional hazard model adjusted for age, sex and number of vaccine doses.

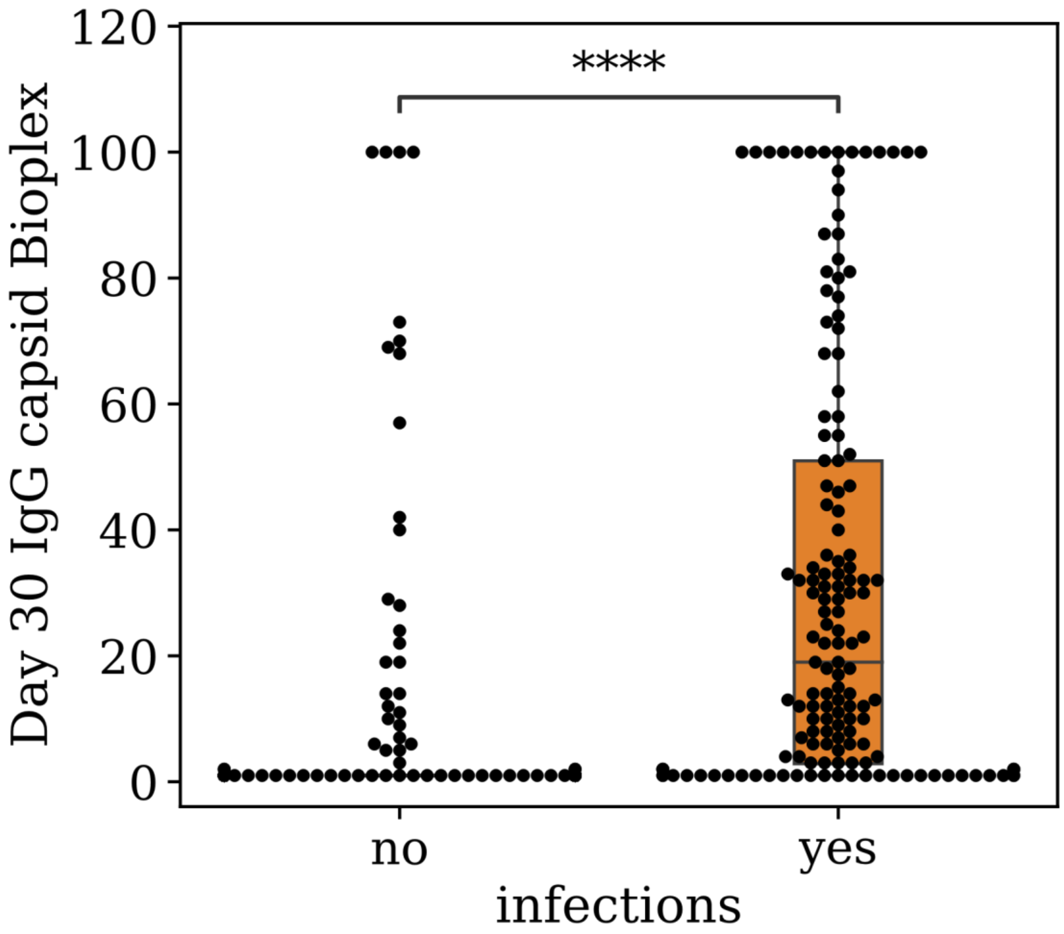

Supplementary Fig. 5: Nucleocapsid IgG antibody levels at day 30. Boxplots of the IgG levels to the nucleocapsid (NC) protein as measured using the Rad Bioplex SARS-CoV-2 assay. The positivity threshold is defined as values > 24. We compared the un-infected (n=452) and infected (n=156) individuals at day 30 of the study. We found that 13 (2.9%) un-infected individuals had positive NC antibody responses suggesting they were asymptomatically infected. Importantly, 82 (52%) infected individuals had no detectable NC antibodies. Black lines represent the median, and boxes indicate the 25th and 75th percentiles. Whiskers represent 1.5 times the interquartile range. P-values were computed using the twosided wilcoxon ranksum test. **** $p < 0.00001$.

Supplementary Table 1.

P-values were computed using a two-sided Cox proportional hazard model estimating vaccine efficacy for 30 follow up days. The model used calendar days as the time-axis and was adjusted for age, occupation, medical center, and time from the third vaccination.

Variable	Hazard Ratio	lower .95	upper .95	P-value
vaccinated Yes	0.55	0.37	0.81	0.002
gender Male	0.98	0.67	1.43	0.91
age grp 35-49	1.15	0.71	1.85	0.576
age grp 50-64	0.8	0.48	1.32	0.382
age grp 65+	0.51	0.19	1.35	0.174
sector of occuppation grp Physicians or Nurses	1.03	0.75	1.43	0.842
hospital Emek	1.16	0.65	2.06	0.61
hospital Meir	0.89	0.42	1.86	0.749
hospital Soroka	1.03	0.61	1.71	0.923
time from third vaccine (months)	1.03	0.83	1.27	0.792

Supplementary Table 2.

P-values were computed using a two-sided Cox proportional hazard model estimating vaccine efficacy at 60-90 follow up days. The model used calendar days as the time-axis and was adjusted for age, occupation, medical center, and time from the third vaccination.

Variable	Hazard Ratio	lower .95	upper .95	P-value
vaccinated Yes	0.63	0.46	0.85	0.003
gender Male	0.88	0.65	1.21	0.439
age grp 35-49	1.06	0.72	1.56	0.755
age grp 50-64	0.76	0.51	1.13	0.174
age grp 65+	0.44	0.2	0.97	0.041
sector of occuppation grp Physicians or Nurses	1	0.77	1.3	0.989
hospital Emek	1.1	0.69	1.76	0.696
hospital Meir	1.16	0.66	2.04	0.597
hospital Soroka	1.06	0.7	1.59	0.792
time from third vaccine (months)	1.06	0.89	1.27	0.525

Supplementary Table 3.

P-values were computed using a two-sided Poisson regression estimating vaccine efficacy for 30 follow up days. The model was adjusted to the daily proportion of positive PCR tests to Covid-19, age, occupation, medical center, and time from the third vaccination and subjects as a random effect.

Variable	Incidence rate ratio	lower .95	upper .95	p-value
vaccinated Yes	0.43	0.28	0.64	<0.001
percent pcr	1.14	1.09	1.18	<0.001
age grp 35-49	1.12	0.68	1.82	0.66
age grp 50-64	0.8	0.47	1.34	0.387
age grp 65+	0.45	0.15	1.33	0.151
hospital Emek	1.28	0.69	2.35	0.431
hospital Meir	1.01	0.47	2.19	0.972
hospital Soroka	1.23	0.7	2.13	0.471
sector of occuppation grp Physicians or Nurses	1.13	0.81	1.59	0.467
time from third vaccine (months)	1.02	0.82	1.25	0.886
gender Male	0.99	0.67	1.47	0.96

Supplementary Table 4.

P-values were computed using a two-sided Poisson regression estimating vaccine efficacy for 60-90 follow up days. The model was adjusted to the daily proportion of positive PCR tests to Covid-19, age, occupation, medical center, and time from the third vaccination and subjects as a random effect.

**Variable**	**Incidence rate ratio**	**lower .95**	**upper .95**	**p-value**
vaccinated Yes	0.55	0.41	0.74	<0.001
percent pcr	1.08	1.06	1.1	<0.001
age grp 35-49	1.08	0.74	1.59	0.683
age grp 50-64	0.77	0.52	1.16	0.21
age grp 65+	0.47	0.21	1.01	0.054
hospital Emek	1.06	0.66	1.7	0.804
hospital Meir	1.12	0.64	1.96	0.686
hospital Soroka	1.08	0.72	1.62	0.714
sector of occuppation grp Physicians or Nurses	1.02	0.79	1.33	0.874
time from third vaccine (months)	1.06	0.89	1.27	0.509
gender Male	0.88	0.64	1.2	0.406

Supplementary Table 5.

P-values were computed using a two-sided Cox proportional hazard model comparing infection hazard at 30 follow up days in the low-baseline with high-baseline response groups, using the five primary analysis baseline markers. The model used calendar days as the time-axis and was adjusted for age, occupation, medical center, and time from the third vaccination.

Marker	Hazard Ratio	lower .95	upper .95	p-value
**Three doses**				
IgG BioPlex S2	1.39	0.95	2.05	0.089
IgG Alinity RBD	1.59	1.07	2.35	0.02
IgG Mutants RBD	1.5	0.87	2.61	0.148
IgA Variants	1.34	0.76	2.37	0.309
IgA Wuhan	1.09	0.63	1.89	0.756
**Four doses**				
IgG BioPlex S2	1.32	0.71	2.45	0.389
IgG Alinity RBD	1.47	0.77	2.78	0.239
IgG Mutants RBD	2.07	0.73	5.83	0.171
IgA Variants	4.45	1.52	13.02	0.006
IgA Wuhan	3.19	1.21	8.38	0.019

Supplementary Table 6.

P-values were computed using a two-sided Cox proportional hazard model comparing infection hazard at 60-90 follow up days in the low-baseline with high-baseline response groups, using the five primary analysis baseline markers. The model used calendar days as the time-axis and was adjusted for age, occupation, medical center, and time from the third vaccination.

Marker	Hazard Ratio	lower .95	upper .95	p-value
**Three doses**				
IgG BioPlex S2	1.45	1.05	1.99	0.022
IgG Alinity RBD	1.54	1.12	2.13	0.008
IgG Mutants RBD	1.52	0.95	2.42	0.079
IgA Variants	1.47	0.92	2.34	0.107
IgA Wuhan	1.33	0.84	2.11	0.217
**Four doses**				
IgG BioPlex S2	1.77	1.07	2.92	0.025
IgG Alinity RBD	1.65	1	2.71	0.049
IgG Mutants RBD	2.24	0.99	5.04	0.053
IgA Variants	2.04	0.96	4.35	0.065
IgA Wuhan	2.05	1.03	4.09	0.041

Supplementary Table 7.

P-values were computed using a two-sided Cox proportional hazard model comparing infection hazard at 30 follow up days in the low-baseline with high-baseline response groups, using all pairwise combinations of the five primary analysis baseline markers. The model used calendar days as the timeaxis and was adjusted for age, occupation, medical center, and time from the third vaccination.

**Marker combination**	**Hazard Ratio**	**lower .95**	**upper .95**	**p-value**
**Three doses**				
IgG Mutants RBD & IgA Variants	4.49	1.07	18.8	0.04
IgG Alinity RBD & IgA Variants	3.45	1.35	8.81	0.01
IgG Alinity RBD & IgG Mutants RBD	1.98	1.05	3.7	0.034
IgG Mutants RBD & IgA Wuhan	2.75	0.74	10.2	0.13
BioPlex IgG S2 & IgG Alinity RBD	1.76	1.11	2.79	0.016
BioPlex IgG S2 & IgG Mutants RBD	1.93	0.99	3.77	0.055
BioPlex IgG S2 & IgA Variants	2.94	1.22	7.07	0.016
BioPlex IgG S2 & IgA Wuhan	1.63	0.72	3.67	0.237
IgA Wuhan & IgA Variants	1.48	0.73	3.02	0.277
IgG Alinity RBD & IgA Wuhan	1.64	0.72	3.76	0.242
**Four doses**				
IgG Mutants RBD & IgA Variants	12.24	1.15	130.11	0.038
IgG Alinity RBD & IgA Variants	4.38	1.23	15.65	0.023
IgG Alinity RBD & IgG Mutants RBD	1.96	0.6	6.42	0.269
IgG Mutants RBD & IgA Wuhan	6.15	0.94	40.38	0.058
BioPlex IgG S2 & IgG Alinity RBD	1.72	0.76	3.9	0.195
BioPlex IgG S2 & IgG Mutants RBD	2.77	0.69	11.16	0.153
BioPlex IgG S2 & IgA Variants	3.04	0.92	10.07	0.068
BioPlex IgG S2 & IgA Wuhan	2.87	0.88	9.38	0.082
IgA Wuhan & IgA Variants	5.73	1.54	21.26	0.009
IgG Alinity RBD & IgA Wuhan	2.51	0.82	7.74	0.108

Supplementary Table 8.

P-values were computed using a two-sided Cox proportional hazard model comparing infection hazard at 60-90 follow up days in the low-baseline with high-baseline response groups, using all pairwise combinations of the five primary analysis baseline markers. The model used calendar days as the time axis and was adjusted for age, occupation, medical center, and time from the third vaccination.

Marker combination	Hazard Ratio	lower .95	upper .95	p-value
**Three doses**				
IgG Mutants RBD & IgA Variants	6.34	1.62	24.86	0.008
IgG Alinity RBD & IgA Variants	3.3	1.5	7.25	0.003
IgG Alinity RBD & IgG Mutants RBD	1.9	1.12	3.22	0.017
IgG Mutants RBD & IgA Wuhan	4.94	1.39	17.58	0.014
BioPlex IgG S2 & IgG Alinity RBD	1.84	1.24	2.72	0.002
BioPlex IgG S2 & IgG Mutants RBD	1.76	1.01	3.07	0.046
BioPlex IgG S2 & IgA Variants	2.88	1.32	6.28	0.008
BioPlex IgG S2 & IgA Wuhan	2.23	1.08	4.59	0.03
IgA Wuhan & IgA Variants	1.58	0.88	2.82	0.123
IgG Alinity RBD & IgA Wuhan	1.95	0.97	3.95	0.062
**Four doses**				
IgG Mutants RBD & IgA Variants	8.14	1.43	46.41	0.018
IgG Alinity RBD & IgA Variants	3.47	1.23	9.84	0.019
IgG Alinity RBD & IgG Mutants RBD	2.27	0.89	5.83	0.088
IgG Mutants RBD & IgA Wuhan	7.67	1.62	36.29	0.01
BioPlex IgG S2 & IgG Alinity RBD	2.01	1.09	3.7	0.025
BioPlex IgG S2 & IgG Mutants RBD	2.7	0.94	7.75	0.066
BioPlex IgG S2 & IgA Variants	2.61	0.95	7.17	0.064
BioPlex IgG S2 & IgA Wuhan	3.29	1.25	8.68	0.016
IgA Wuhan & IgA Variants	2.34	1	5.48	0.051
IgG Alinity RBD & IgA Wuhan	2.91	1.12	7.6	0.029

Supplementary Table 9.

P-values were computed using a two-sided Poisson regression comparing infection incidence at 30 follow up days, of the low-baseline and high-baseline response groups using the five primary analysis baseline markers. The model was adjusted to the daily proportion of positive PCR tests to Covid-19, age, occupation, medical center, and time from the third vaccination and subjects as a random effect.

Marker	Incidence rate ratio	lower .95	upper .95	p-value
**Three doses**				
IgG BioPlex S2	1.36	0.93	2	0.118
IgG Alinity RBD	1.53	1.03	2.27	0.033
IgG Mutants RBD	1.42	0.81	2.48	0.222
IgA Variants	1.3	0.74	2.27	0.363
IgA Wuhan	1.05	0.6	1.83	0.859
**Four doses**				
IgG BioPlex S2	1.19	0.6	2.36	0.619
IgG Alinity RBD	1.44	0.48	4.27	0.516
IgG Mutants RBD	1.57	0.56	4.46	0.393
IgA Variants	4.53	1.22	16.82	0.024
IgA Wuhan	2.71	0.91	8.05	0.073

Supplementary Table 10.

P-values were computed using a two-sided Poisson regression comparing infection incidence at 60-90 follow up days, of the low-baseline and high-baseline response groups using the five primary analysis baseline markers. The model was adjusted to the daily proportion of positive PCR tests to Covid-19, age, occupation, medical center, and time from the third vaccination and subjects as a random effect.

Marker	Incidence rate ratio	lower .95	upper .95	p-value
**Three doses**				
IgG BioPlex S2	1.46	1.06	2	0.02
IgG Alinity RBD	1.56	1.13	2.15	0.007
IgG Mutants RBD	1.49	0.93	2.38	0.096
IgA Variants	1.38	0.87	2.18	0.173
IgA Wuhan	1.25	0.79	1.96	0.335
**Four doses**				
IgG BioPlex S2	1.69	1.03	2.76	0.037
IgG Alinity RBD	1.67	1.02	2.74	0.04
IgG Mutants RBD	2.2	0.99	4.89	0.052
IgA Variants	1.82	0.87	3.8	0.11
IgA Wuhan	1.88	0.95	3.69	0.069

Supplementary Table 11.

P-values were computed using a two-sided Poisson regression comparing infection incidence at 30 follow up days of the low-baseline and high-baseline response groups using all pairwise combinations of the five primary analysis baseline markers. The model was adjusted to the daily proportion of positive PCR tests to Covid-19, age, occupation, medical center, and time from the third vaccination and subjects as a random effect.

**Marker combination**	**Incidence rate ratio**	**lower .95**	**upper .95**	**p-value**
**Three doses**				
IgG Mutants RBD & IgA Variants	2.92	0.75	11.27	0.121
IgG Alinity RBD & IgA Variants	2.86	1.16	7.08	0.023
IgG Alinity RBD & IgG Mutants RBD	1.83	0.97	3.44	0.062
IgG Mutants RBD & IgA Wuhan	2.64	0.69	10.06	0.155
BioPlex IgG S2 & IgG Alinity RBD	1.68	1.06	2.66	0.028
BioPlex IgG S2 & IgG Mutants RBD	1.76	0.89	3.47	0.101
BioPlex IgG S2 & IgA Variants	2.58	1.09	6.09	0.031
BioPlex IgG S2 & IgA Wuhan	1.52	0.66	3.46	0.323
IgA Wuhan & IgA Variants	1.39	0.69	2.82	0.359
IgG Alinity RBD & IgA Wuhan	1.53	0.66	3.53	0.319
**Four doses**				
IgG Mutants RBD & IgA Variants	5.48	0.51	59.07	0.16
IgG Alinity RBD & IgA Variants	4.71	1.12	19.76	0.034
IgG Alinity RBD & IgG Mutants RBD	1.3	0.36	4.64	0.688
IgG Mutants RBD & IgA Wuhan	3.63	0.59	22.24	0.163
BioPlex IgG S2 & IgG Alinity RBD	1.43	0.58	3.54	0.443
BioPlex IgG S2 & IgG Mutants RBD	1.61	0.35	7.35	0.536
BioPlex IgG S2 & IgA Variants	2.14	0.52	8.77	0.29
BioPlex IgG S2 & IgA Wuhan	1.89	0.51	6.99	0.339
IgA Wuhan & IgA Variants	4.49	0.92	21.78	0.063
IgG Alinity RBD & IgA Wuhan	2.08	0.61	7.09	0.244

Supplementary Table 12.

P-values were computed using a two-sided Poisson regression comparing infection incidence at 60-90 follow up days of the low-baseline and high-baseline response groups using all pairwise combinations of the five primary analysis baseline markers. The model was adjusted to the daily proportion of positive PCR tests to Covid-19, age, occupation, medical center, and time from the third vaccination and subjects as a random effect.

Marker combination	Incidence rate ratio	lower .95	upper .95	p-value
**Three doses**				
IgG Mutants RBD & IgA Variants	4.66	1.29	16.87	0.019
IgG Alinity RBD & IgA Variants	2.96	1.38	6.35	0.005
IgG Alinity RBD & IgG Mutants RBD	1.85	1.09	3.13	0.022
IgG Mutants RBD & IgA Wuhan	4.5	1.28	15.87	0.019
BioPlex IgG S2 & IgG Alinity RBD	1.85	1.25	2.73	0.002
BioPlex IgG S2 & IgG Mutants RBD	1.68	0.96	2.93	0.07
BioPlex IgG S2 & IgA Variants	2.55	1.19	5.44	0.016
BioPlex IgG S2 & IgA Wuhan	2.22	1.08	4.58	0.031
IgA Wuhan & IgA Variants	1.47	0.83	2.61	0.189
IgG Alinity RBD & IgA Wuhan	1.98	0.98	4	0.058
**Four doses**				
IgG Mutants RBD & IgA Variants	6.87	1.26	37.38	0.026
IgG Alinity RBD & IgA Variants	3.33	1.21	9.16	0.02
IgG Alinity RBD & IgG Mutants RBD	2.24	0.9	5.55	0.083
IgG Mutants RBD & IgA Wuhan	6.45	1.4	29.6	0.017
BioPlex IgG S2 & IgG Alinity RBD	1.99	1.08	3.66	0.027
BioPlex IgG S2 & IgG Mutants RBD	2.61	0.91	7.48	0.075
BioPlex IgG S2 & IgA Variants	2.35	0.9	6.13	0.08
BioPlex IgG S2 & IgA Wuhan	2.85	1.14	7.15	0.025
IgA Wuhan & IgA Variants	2.07	0.9	4.73	0.086
IgG Alinity RBD & IgA Wuhan	2.93	1.11	7.72	0.03

Supplementary Table 13.

Demographic characteristics of the Validation cohort participants

	Overall
N	46
Sex, Male (%)	18
Age (mean (SD))	28
Age group (%)	
18-34	30
35-49	14
50-64	1
65+	1
Number of vaccination doses	
3 doses	41
4 doses	5

Supplementary Table 14.

P-values were computed using a two-sided Cox proportional hazard model comparing infection hazard at 198 follow up days in the low-baseline with high-baseline response groups, using all pairwise combinations of the four primary analysis baseline markers. The model used calendar days as the timeaxis and was adjusted for age, sex and number of vaccine doses.

Marker	Hazard Ratio	Confidence interval	p-value
IgA Wuhan	2.02	0.69 – 5.94	0.199
IgG Wuhan	3.96	1.23 – 12.73	0.021
IgA Variants	1.62	0.55 – 4.72	0.38
IgG Variants	3.56	1.24 – 10.25	0.019
IgG Wuhan and IgA Wuhan	2.96	0.63 – 13.83	0.168
IgG Variants and IgA Variants	4.14	1.11 – 15.36	0.034
IgG Wuhan and IgA Variants	5.05	1.25 – 20.43	0.023
IgG Variants and IgA Wuhan	8.5	1.81 – 39.81	0.007

Supplementary Table 15.

P-values were computed using a two-sided Cox proportional hazard model comparing infection hazard at 290 follow up days in the low-baseline with high-baseline response groups, using all pairwise combinations of the four primary analysis baseline markers. The model used calendar days as the timeaxis and was adjusted for age, sex and number of vaccine doses.

Marker	Hazard Ratio	Confidence interval	p-value
IgA Wuhan	2.84	1.07 – 7.55	0.036
IgG Wuhan	4.18	1.48 – 11.78	0.007
IgA Variants	1.57	0.59 – 4.17	0.368
IgG Variants	3.74	1.45 – 9.64	0.006
IgG Wuhan and IgA Wuhan	3.74	1.45 – 9.64	0.006
IgG Variants and IgA Variants	5.06	1.50 – 17.09	0.009
IgG Wuhan and IgA Variants	6.59	1.66 – 26.20	0.007
IgG Variants and IgA Wuhan	8.62	2.15 – 34.49	0.002

Supplementary Table 16.

Numbers of individuals per vaccine group is for each pairwise combination markers.

	**Three doses**	**Four doses**
IgG Mutant RBD & IgA Variants	52	42
IgG Mutant RBD & IgA Wuhan	51	46
IgG Alinity RBD & IgA Variants	90	71
IgG BioPlex S2 & IgA Wuhan	93	78
IgG BioPlex S2 & IgA Variants	88	75
IgG Alinity RBD & IgA Wuhan	105	78
IgG BioPlex S2 & IgG Mutant RBD	122	77
IgG Alinity RBD & IgG Mutant RBD	144	101
IgG Alinity RBD & IgG BioPlex S2	259	172
IgA Wuhan & IgA Variants	116	84
