# Supplementary material for: Correlates of protection for booster doses of the SARS-CoV-2 vaccine BNT162b2
Source: Nat Commun. 2023 Jul 29;14:4575. doi: 10.1038/s41467-023-39816-4 (PMC10387073; doi:10.1038/s41467-023-39816-4)
Supplement: Supplementary file 3 — Description of Additional Supplementary Files [file 41467_2023_39816_MOESM3_ESM.pdf]

Description of Additional Supplementary Files

Supplementary Data 1: Experimental data including antigen microarray, ELISA and neutralization assays measurements as well as infection and vaccination information for each individual.

Supplementary Data 2: Control cohort (Supplementary Fig. 1) antigen microarray area under the curve results and information about SARS-CoV-2 status.

Supplementary Data 3: The subset of 74 individuals in the immunogenicity subsets that were selected at baseline and belong to the low-baseline (n=38) and high-baseline (n=36) immune history quartiles.

Supplementary Data 4: Demographics, vaccination status and infection status of the 74 individuals in the immunogenicity subset used in the study, which included 38 low-baseline and 36 high-baseline individuals. P-values were computed using a t-test was used to compare the continuous variables and chi-square test was used for categorical variables.

Supplementary Data 5: list of antigens spotted on the SARS-CoV-2 antigen microarrays.

Supplementary Data 6: Hazard ratios and p-values of a Cox proportional hazard model for different baseline makers computed for invidious that received three doses of the Pfizer vaccine. P-values were computed using a two-sided Cox proportional hazard model comparing infection hazard at 60-90 follow up days in the low-baseline with high-baseline response groups using all markers. We used calendar days as the time-axis and adjusted for age, occupation, medical center, and time from the third vaccination. Results are presented in a decreasing order based on HR value and significant p-values are colored in bold.

Supplementary Data 7: Hazard ratios and p-values of a Cox proportional hazard model for different baseline makers computed for invidious that received four doses of the Pfizer vaccine. P-values were computed using a two-sided Cox proportional hazard model comparing infection hazard at 60-90 follow up days in the low-baseline with high-baseline response groups using all markers. We used calendar days as the time-axis and adjusted for age, occupation, medical center, and time from the third vaccination. Results are presented in a decreasing order based on HR value and significant p-values are colored in bold.

Supplementary Data 8: Baseline immune history groupings of the validation cohort using different baseline markers.
